# Characterization of Wastewater Treatment Plant Microbial Communities and the Effects of Carbon Sources on Diversity in Laboratory Models

**DOI:** 10.1371/journal.pone.0105689

**Published:** 2014-08-22

**Authors:** Sangwon Lee, Jil T. Geller, Tamas Torok, Cindy H. Wu, Mary Singer, Francine C. Reid, Daniel R. Tarjan, Terry C. Hazen, Adam P. Arkin, Nathan J. Hillson

**Affiliations:** 1 Physical Biosciences Division, Lawrence Berkeley National Laboratory, Berkeley, California, United States of America; 2 Earth Sciences Division, Lawrence Berkeley National Laboratory, Berkeley, California, United States of America; University of Wisconsin, Food Research Institute, United States of America

## Abstract

We are developing a laboratory-scale model to improve our understanding and capacity to assess the biological risks of genetically engineered bacteria and their genetic elements in the natural environment. Our hypothetical scenario concerns an industrial bioreactor failure resulting in the introduction of genetically engineered bacteria to a downstream municipal wastewater treatment plant (MWWTP). As the first step towards developing a model for this scenario, we sampled microbial communities from the aeration basin of a MWWTP at three seasonal time points. Having established a baseline for community composition, we investigated how the community changed when propagated in the laboratory, including cell culture media conditions that could provide selective pressure in future studies. Specifically, using PhyloChip 16S-rRNA-gene targeting microarrays, we compared the compositions of sampled communities to those of inocula propagated in the laboratory in simulated wastewater conditionally amended with various carbon sources (glucose, chloroacetate, D-threonine) or the ionic liquid 1-ethyl-3-methylimidazolium chloride ([C2mim]Cl). *Proteobacteria*, *Bacteroidetes*, and *Actinobacteria* were predominant in both aeration basin and laboratory-cultured communities. Laboratory-cultured communities were enriched in *γ-Proteobacteria*. *Enterobacteriaceae*, and *Aeromonadaceae* were enriched by glucose, *Pseudomonadaceae* by chloroacetate and D-threonine, and *Burkholderiacea* by high (50 mM) concentrations of chloroacetate. Microbial communities cultured with chloroacetate and D-threonine were more similar to sampled field communities than those cultured with glucose or [C2mim]Cl. Although observed relative richness in operational taxonomic units (OTUs) was lower for laboratory cultures than for field communities, both flask and reactor systems supported phylogenetically diverse communities. These results importantly provide a foundation for laboratory models of industrial bioreactor failure scenarios.

## Introduction

In recent years, there have been increasing numbers of genetically engineered microorganisms (GEMs) constructed for biotechnology, medicine, bioenergy, bio-based chemicals and materials, and bioremediation applications [1]–[4]. While containment strategies including engineered auxotrophy, induced lethality, and gene-flow barriers have been developed to mitigate the environmental risks posed by GEMs, naturally occurring processes may negate these genetic safeguards [5]. Currently, we cannot confidently predict how well a given GEM will survive and adapt if released into a given environment, nor how it will impact ecological biodiversity or interact with indigenous microbes (*e.g.*, horizontal gene transfer and subsequent phenotype alteration [6], [7]).

A potential scenario to consider is an industrial bioreactor failure that results in the exposure of GEMs to the microbial communities indigenous to a downstream municipal wastewater treatment system.

Such a wastewater treatment system typically performs primary and secondary biological treatment (activated sludge) of municipal wastewater before release, and possibly tertiary treatment (microfiltration) for applications including landscape and agricultural irrigation, industrial processes, and wetlands restoration. Domestic sewage and other wastewater received by the treatment plant first undergo primary sedimentation. Effluent from the primary sedimentation basins flows to aeration basins where divers microorganisms (originating from the received wastewater) consume the soluble organic matter. Effluent from the aeration basins flows to clarifiers, which sediment the microorganisms from the water. The clarifier effluent is either discharged or further processed for re-use. The clarifier sediment, or “activated sludge”, is recycled back to the aeration basins to maintain a high cell density of microorganisms and removal rates of soluble organic matter, while the net growth of microorganisms is sent to anaerobic digesters, which also receive primary sedimentation sludge. The material of the aeration basin is referred to as “mixed liquor”, reflecting the mixture of the recycled activated sludge and the inflow to the aeration basin. The aeration basin contains a complex and high-density microbial community, which could serve as a hot spot for horizontal gene transfer.

While a given laboratory GEM itself may be unlikely to survive in an aeration basin, the portion, if any, of the GEM's genetic material that confers advantage to others in the microbial community (post horizontal gene transfer) may have a greater chance for survival. Our long-term primary questions of interest (only partially resolved by the work reported herein), then, are which selectable capabilities found within GEM genetic material might provide sufficient advantage to be effectively transferred to other members of the community, how the composition of the community would be affected by gene transfer, and how is this transfer limited by environmental constraints.

Efforts to characterize microbial structure and gene expression within a Hong Kong MWWTP activated sludge community [8], to investigate metagenomic determinants of reduced antimicrobial drug susceptibility within MWWTP bacteria [9], [10], and to propagate MWWTP microbial communities in replicate membrane bioreactors [11] in the presence of the model toxin 3-chloroaniline [12], have all been recently reported. In order to further advance our understanding of potential gene transfer from GEMs to a diverse microbial community representative of a municipal wastewater treatment plant (MWWTP), it is important to establish suitable selection conditions (media and genetic markers) for the gene transfer, and to characterize the baseline microbial communities resulting from each condition. To this end, we inventoried and compared aeration-basin bacterial communities and laboratory-grown communities, which were cultured with various carbon sources using preserved and maintained mixed-liquor communities as inoculum. For the laboratory model system, we selected carbon sources as selective media components that promote the activity of carbon catabolic genes. These selective markers, which could be overexpressed in GEMs in future experiments were hypothesized to be rare in MWWTP microbial communities. For example, we selected the ionic liquid 1-ethyl-3-methylimidazolium chloride ([C2mim]Cl), a microbial growth inhibitor and deconstruction agent for lignocellulosic feedstocks for biofuels-producing GEMs [13], as a selective medium that promotes the activity of rare efflux-pump tolerance genes. An efflux pump for [C2mim]Cl was recently identified in *Enterobacter lignolyticus* SCF1 and cross-validated in *E. coli* [14], which is evidence that horizontal gene transfer could confer the [C2mim]Cl tolerance phenotype. We also screened 192 carbon sources employing the phenotype microarray, OmniLog PM MicroPlate (BioLog, Hayward, CA) and identified carbon sources, including D-threonine, that neither MWWTP microbial communities nor *E. coli* (a potential parental lineage for GEMs) appeared to metabolize well, showing very poor growth compared to other carbon sources (data not shown). Note that the selected carbon sources, including D-threonine, chloroacetate and [C2mim]Cl, were not chosen because they are likely be found in (or especially relevant to) MWWTPs, but rather because they could potentially serve well as selective media components for future laboratory experiments. We hypothesize that the selected carbon sources will prove more effective than antimicrobial drugs as selective agents for future laboratory experiments since resistance genes for various antimicrobial drugs already appear to be prevalent in activated sludge microbial communities [9], [10].

In this study, we have used PhyloChip G3, a third generation high-density phylogenetic DNA microarray that has been successfully employed in microbial ecology [15]–[18] to investigate microbial community structure.

## Materials and Methods

### Overall experimental design

**Figure 1:**
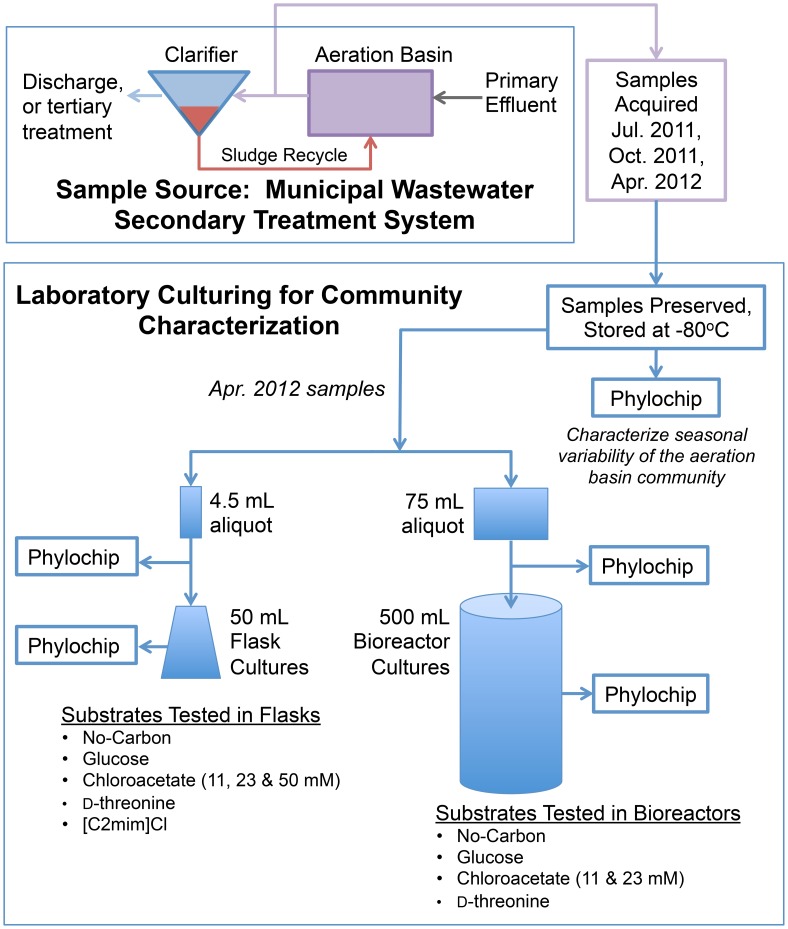
Overview of Experiments. All substrates were tested in triplicate, except for ‘No-Carbon in bioreactors’, which was tested in duplicate. The No-Carbon and [C2mim]Cl triplicates in flasks were pooled in order to provide adequate biomass for PhyloChip analysis.

An overview of the experiments conducted herein is schematically depicted in Fig. 1.

### Aeration basin samples

Samples of mixed liquor from the secondary treatment unit of a local San Francisco Bay Area wastewater treatment plant with typical secondary processes that discharges into San Francisco Bay were obtained from the channel that flows from the aeration basins to the clarifiers. (John R. Cloak, who is authorized to give all required permission and should be contacted for future permission, facilitated sample acquisition on private land and provided plant operation data. Sample acquisition did not involve endangered or protected species.) On three sampling dates (July 20, 2011, October 19, 2011, and April 25, 2012), four sterile 1-L bottles were each filled with 500 mL of sample and transported at ambient temperature to the laboratory for cryo-preservation as inoculum for subsequent culturing and PhyloChip characterization (described below). Inoculum was preserved by adding 1.5 mL of sterile 45% (w/v) glycerol solution to 3 mL of sample, and then storing it at −80°C. Larger 75 mL aliquots (50 mL sample with 25 mL 45% (w/v) glycerol solution for bioreactor inoculation) were also preserved from the April 25, 2012 samples. Glycerol stock aliquots were prepared under constant stirring to mitigate sludge microbe flocculation and maintain homogenous samples.

### Laboratory cultures

Batch cultures were grown at 26°C in baffled Erlenmeyer flasks (50 mL culture in 250-mL flask shaken at 250 rpm on an orbital shaker) and bioreactors (500 mL custom-manufactured vessels used with controllers manufactured by Fairmentec GmbH, Göttingen, Germany, Bioreactor RK01-40). Each liter of the defined basal medium contained 0.87 g K_2_HPO_4_, 0.88 g KH_2_PO_4_, 5.5 mg CaCl_2_, 0.11 g NH_4_Cl, 31 mg CH_4_N_2_O, 0.2 g MgSO_4_•7H_2_O, 2.4 mg C_6_H_5_FeO_7_, 25 mg H_3_BO_3_, 2.9 mg ZnSO_4_•7H_2_O, 0.6 mg KI, 2.5 mg CuSO_4_•5H_2_O, 8.4 mg CoSO_4_•7H_2_O, 0.8 mg Na_2_MoO_4_•2H_2_O, 16 mg MnCl_2_•4H_2_O, and 63 mg N(CH_2_COONa)_3_ (nitrilotriacetic acid trisodium salt). The bioreactors were aerated with sterile filtered (0.2 µm) air through glass frits, pH controlled to 6.8 with 4% HCl and 20 g/L Na_2_CO_3_ and agitated by means of a magnetic stir bar at 400 rpm. Dissolved oxygen monitoring of the bioreactors indicated that oxygen levels remained at saturation throughout growth. The reactors and flasks were inoculated to an initial OD_600 nm_ of 0.05, using the preserved April 25, 2012 samples, which were washed three times with the basal medium to remove residual glycerol before inoculation.

Carbon sources were added to the basal medium to provide an equivalent carbon concentration of 0.26 g C/L, to approximate the range of carbonaceous biochemical oxygen demand values of the primary sedimentation effluent at the wastewater treatment plant. Glucose was selected as a carbon source that could potentially be metabolized by a broad range of microorganisms. D-threonine (5.5 mM), chloroacetate (11 mM), as well as the ionic liquid [C2mim]Cl (270 mM) were used as candidates for selective carbon sources. Chloroacetate was also tested at higher concentrations (23 mM and 50 mM) that would potentially be growth inhibitory to the aeration-basin microorganisms. As controls, cultures were grown in basal medium without any additional carbon source (no-carbon). Negative controls in glucose-amended basal medium were also performed in the flasks.

Growth of the batch cultures was monitored by taking samples for OD_600 nm_ and substrate concentration measurement. In the reactors, OD_600 nm_ was continuously monitored by pumping a recycle line through a cuvette in a small spectrophotometer (USB400, Ocean Optics, FL). Samples for PhyloChip were taken once cultures reached stationary phase, or immediately following the peak OD_600 nm_. All conditions were tested in triplicate, with the following exceptions: one negative-control was run per batch of flask tests, and two no-carbon reactor tests were performed.

### DNA extraction and PCR for microarray analyses

Microbial community genomic DNA was extracted from three biological replicates per sample whenever available. For each aeration-basin sample, a 4.5 mL stock vial containing 3 mL sample and 1.5 mL of sterile 45% (w/v) glycerol was considered a biological replicate and gDNA was extracted from three stock vials per sample-date. For laboratory-cultured samples, three flask-, or three reactor-cultures per each treatment were used for gDNA extraction. When the microbial cell mass was very low ([C2mim]Cl, and no-carbon flask control cultures), the three biological replicates were pooled to obtain sufficient amount of gDNA.

Biomass for gDNA extraction was concentrated either by centrifugation for the aeration-basin samples, or by filtration through Sterivex GP filter units with 0.22 µm pore size (Millipore; Billerica, MA) for the laboratory cultures. Sample was pushed through the filter with a syringe until no more filtrate would pass. The amount of samples filtered ranged from 5 to 60 mL, depending upon the cell density of the sample. The filters were aseptically removed from the filter units and placed into separate sterile 1.5 mL microcentrifuge tubes and stored overnight at 4°C in RNA*later* solution (Ambion; Austin, TX). On the following day, the RNA*later* solution was removed and the filters were stored at −80°C until further processing.

A DNA-EZ Kit (GeneRite; North Brunswick, NJ) was used for gDNA extraction following the manufacturer's instruction enhanced by bead beating. Briefly, filters were cut aseptically and each filter piece, or one third of the pellet, was placed in Lysing Matrix E tubes (MP Biomedicals; Solon, OH) with 500 µL of the kit's lysing buffer and treated in a FastPrep bead beater (Savant; Carlsbad, CA) at 5.0 m/s for 45 s twice. The lysed solution was applied to a DNA binding column. After several washes, gDNA was eluted with 100 µL of elution buffer. The eluted sub-samples were pooled and the gDNA quantified using a Qubit dsDNA HS Assay Kit (Invitrogen, Grand Island, NY).

Bacterial 16S rRNA genes were amplified with primers 27F (5′-AGAGTTTGATCCTGGCTCAG-3′) and 1492R (5′-GGTTACCTTGTTACGACTT-3′). Each PCR reaction contained 1×Ex Taq buffer (Takara Bio Inc., Japan), 0.025 units/µL ExTaq polymerase, 200 mM dNTP mixture, 1.0 µg/µL BSA, 200 nM each primer, and 1 ng genomic DNA. The temperature gradient PCR conditions were 95°C (3 min), followed by 25 cycles at 95°C (30 s), 48–58°C (30 s), and 72°C (2 min), and a final extension at 72°C (10 min). Each sample was amplified in 8 replicate 25-µL reactions (one tube per temperature). Amplicons from the gradient amplification were pooled, purified with the QIAquick PCR purification kit (Qiagen; Valencia, CA), and then quantitated using gel electrophoresis (2% agarose E-gel; Invitrogen).

### Microarray processing and data treatment

PhyloChip assay was conducted as previously reported [19] with the following modifications: (1) TDT enzyme (P/N: PM1875; Promega; Madison, WI) and GeneChip Labeling Reagent (P/N: 90042; Affymetrix; Santa Clara, CA) were used for the labeling reaction, and (2) 2.2 µL of 3 nM Control Oligo B2 (Affymetrix) was used for the hybridization reaction. In brief, 500 ng of purified PCR product was fragmented to 50–200 bp, labeled with biotin, hybridized onto the microarray, stained and washed according to the manufacturers instructions. Fluorescent images were scanned with Affymetrix GeneChip Scanner 3000 7G. Probe intensities were background-subtracted and scaled to the quantitative standards. The hybridization score for an OTU was calculated as the mean intensity of the perfectly matching probes excluding the maximum and minimum intensity values. Technical replication of the PhyloChip was not analyzed in this study, since high reproducibility of the PhyloChip has been reported [19].

On the PhyloChip, the average number of probe pairs assigned to each OTU was 37 (s.d. 9.6) [19]. Probe pairs comprised one perfectly matching (PM) 25-mer probe and one mismatching (MM) 25-mer probe, containing a substitution at the central base. The process for scoring an OTU as present/absent occurs in two stages of analysis. Stage 1 of the analysis had the following requirements. (1) There was a minimum of seven probe pairs scored. (2) At least seven probe pairs that were designed for a given OTU were scored positive. Probe pairs were positive if the PM probes had higher intensities than the corresponding MM probes. (3) Ranked response scores (*r* scores) of probe pairs in Quartile_1 were ≥0.8, Quartile_2≥0.93, and Quartile_3≥0.98. The *r* score was calculated based on the A and T count in the probe sequences, and it measures the potential that a probe pair was responding to a target and not the background (see the reference [19]). (4) The OTU probe set had a positive fraction ≥0.92 (positive fraction is the fraction of probe pairs that scored positive in a given OTU). An OTU passing all these criteria passed for consideration at Stage 2. For Stage 2 of the analysis, the cross-hybridization potential of the remaining OTU was determined. Cross-hybridization adjusted response scores (*rx*) were calculated in order to account for potential false positive OTUs, which can occur when a sequence in a sample matches some probes used in multiple, closely related OTU (for the equations, see the reference [19]). The cutoffs for adjusted *rx* scores for calling species present was: Q_1≥0.22, Q_2≥0.40, and Q_3≥0.42. The presence/absence (binary) data of species passing these criteria, the OTU binary data (for OTU belonging to the species called present as long as they passed Stage 1 criteria), and the fluorescence intensities for those OTUs were used for analyzing community structures. An OTU was considered present if it was called present in at least two of the three sample replicates. Taxa at higher levels (phylum, class, order, family, and genus) were considered present if at least one species was called present in a given lineage.

### Community comparison using hierarchical clustering, MRPP and indicator analyses of the relative abundance of OTUs

To investigate similarities between the samples, hierarchical clustering analysis was performed based on the relative abundance of OTUs using PC-ORD (v.6.12; MjM Software, Gleneden Beach, OR). In the analysis, distance was measured by Bray-Curtis and group average was used as a group linkage method. All relative intensity data across the entire sample set was included in the analysis as long as an OTU was called as present in at least one sample. Raw microarray intensity data were imported into PC-ORD, standardized by general relativization by columns (samples), and subsequently transformed by arcsine square root to convert fluorescence intensity data with an arbitrary unit to the numbers that are closer approximations of amplicon amounts used for microarray hybridization.

Multi Response Permutation Procedure (MRPP) in PC-ORD was used to examine the differences among particular *a priori* groups, which were resolved at a particular level of cluster by hierarchical clustering analysis employing Bray-Curtis distance measurement. MRPP is non-parametric procedure, which does not require distributional assumptions, so it is suitable for analyzing ecological community data. MRPP tests the null hypothesis of no difference between within-group distances and across-group distances [20]–[22]. It calculates *δ*, which is a weighted pairwise distance within each group and determines the probability (*p*) value of *δ* being equal, or smaller. It also generates the *A* statistic value, which indicates chance corrected within-group agreement [*A* = 1- (observed *δ*/ expected *δ*)]. If all items are identical within groups (*δ* = 0), then *A* = 1, if heterogeneity within groups equals expectation by chance, then *A* = 0, and if heterogeneity within groups higher than expectation by chance, then *A*<0. As *A* value approaches to 1, pre-defined groups are more similar each other.

To determine which OTUs were responsible for the differences among the seasonal field samples, or among the laboratory-grown communities cultured with various carbon sources, indicator species analysis was conducted following the method by Dufrene and Legendre [23] using PC-ORD. The analysis calculates indicator values of each species (or, OTU) for a particular group by considering both the abundance of the species in a specific group and the faithfulness of occurrence of the species in members of the group. The equation for the indicator value of species *i* in group *j* is as follows: INDVAL *_ij_* = A*_ij_*×B*_ij_*×100. A*_ij_* is a measure of specificity and is calculated by dividing the mean number of species *i* across the members of group *j* by the sum of the mean numbers of species *i* over all groups. B*_ij_* is a measure of fidelity and is obtained by dividing the number of members in group *j*, where species *i* is present, by the total number of members in that group. B*_ij_* is maximized when species *i* is present in all members of group *j*. When we compared the MWWTP seasonal field communities in this study, “group” corresponds to community (for example, the April 25, 2012 sample) and “members” corresponds to the three replicates of that community. The statistical significance of indicator values was tested by Monte Carlo randomization with 1,000 permutations. The indicator analysis was performed at a particular cluster level resolved by hierarchical clustering analysis. The relative abundances of indicator OTUs were visualized by R (version. 2.15.1; Institute for Statistics and Mathematics, Wirtschaftsuniversität Wien [http://www.r-project.org]) [24]. For the R heat-plot, microarray intensities of individual OTUs were centered and scaled by subtracting the mean of the row (OTUs) across all samples and then dividing the resulting values by the standard deviation of the row. The Z scores in heat-plots generated by R represent the distances between the scaled microarray intensities of individual OTUs and the mean of the scaled OTUs. Note that the heat-maps generated by this method are applicable only for comparing OTUs across samples, but not for comparing among OTUs within samples. Average linkage methods were used for hierarchical clustering by R.

### Examination of phylogenetic dissimilarities among the field-sampled and laboratory-grown communities using Principal Coordinate Analysis (PCoA)

A representative 16S rDNA sequence for each OTU was obtained from the GreenGenes database and aligned by the NAST aligner (Lawrence Berkeley National Laboratory [http://greengenes.lbl.gov/cgi-bin/nph-export_records.cgi]) [25], [26]. The filtering criteria for the sequences were: a minimum base number of 1,250 and a maximum non-ACGT character count in the sequences of 50. The alignment comprised the sequences of 7,799 OTUs, which were detected in at least one sample. Using FastTree (v.2.1.7; Lawrence Berkeley National Laboratory [http://www.microbesonline.org/fasttree]) [27], a phylogenetic tree was constructed from the multiple sequence alignment employing the generalized time reversible (GTR) models of nucleotide evolution for distance measurements. FastTree infers approximately-maximum-likelihood phylogenetic trees. FastTree uses a heuristic variant of neighbor joining to get a rough tree topology first, corrects branch lengths using a mix of nearest-neighbor interchanges and subtree- prune-regraft moves, and then improves topology and branch lengths by maximum likelihood rearrangements. Principal Coordinate Analysis (PCoA) was performed using Fast UniFrac (Department of Chemistry and Biochemistry, University of Colorado at Boulder [http://unifrac.colorado.edu])[28] and the PCoA result was plotted using SigmaPlot (v.10; Systat Software Inc., San Jose, CA). A UniFrac distance matrix was calculated from the tree considering within sample normalized abundance weights. In previous PhyloChip validation experiments, the concentrations of PCR amplicons were shown to correlate with the log2 values of the fluorescence intensities with the correlation coefficient (*r*) of 0.917 [29]; therefore, we used the log2 values of the fluorescence intensity scores as the abundance weight.

### Construction of a phylogenetic tree and visualization of the microbial composition of the April 25, 2012 field-sampled community and the laboratory-cultured communities

A single OTU per family was chosen from the GreenGenes database to represent each family in a phylogenetic tree (Table S1). Non-chimeric 16S rDNA sequences of 120 OTUs were obtained from the Hugenholtz 7,682 character data set at GreenGenes and aligned by NAST aligner of GreenGenes as described above. An approximate-maximum-likelihood phylogenetic tree was constructed from the multiple sequence alignment using FastTree as described above and the tree was visualized by iTOL (Interactive Tree Of Life) [30]. An archaeal sequence (DQ300318.1) was included in the tree for rooting.

### Accession reference for PhyloChip data

All PhyloChip data are available for download from the GreenGenes database [26] [http://greengenes.lbl.gov/Download/Microarray_Data/MIAME_data_files_Wastewater-reactors_Hillson.zip]. All PhyloChip data were deposited in NCBI's Gene Expression Omnibus [31] and are accessible through GEO Series accession number GSE58251 [http://www.ncbi.nlm.nih.gov/geo/query/acc.cgi?acc=GSE58251].

## Results

### Microbial communities detected in the aeration-basin samples

**Figure 2:**
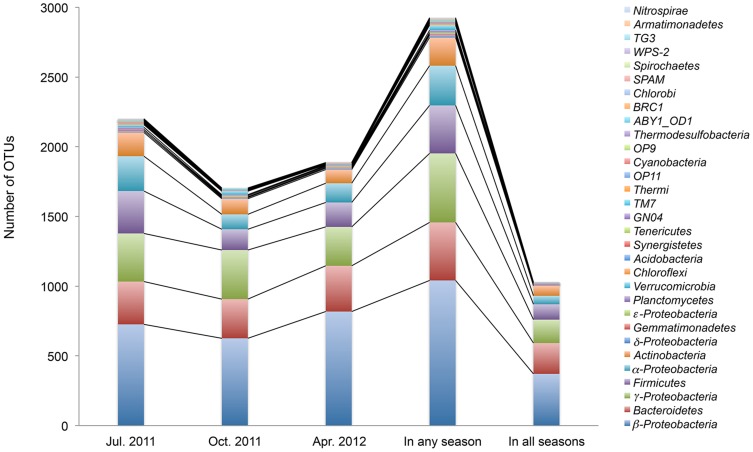
Seasonal composition of aeration-basin bacterial community samples, as represented by the number of OTUs. The composition is shown at the phylum level, or at the class level for the phylum *Proteobacteria*. An OTU was counted present if it was detected in at least two of three sample replicates.

A species was considered present if it was called present in at least two of the three sample replicates. A phylum was considered present if at least one species was called present in a given phylum (see the Materials and methods for details). The July 20, 2011 sample was the most diverse community consisting of 24 phyla, 234 species, and 2,200 OTUs (Fig. 2). The October 19, 2011 sample contained 17 phyla, 192 species, 1,704 OTUs, and the April 25, 2012 sample had 16 phyla, 182 species and 1,886 OTUs. The numbers of the OTUs at phylum level (or, class level for the major phyla) detected in the seasonal samples are listed in the Table S2. The majority (96%–98%) of OTUs in each sample belong to four phyla: *Proteobacteria*, *Bacteroidetes*, *Firmicutes*, and *Actinobacteria*. While probes that target these four phyla are overrepresented on the PhyloChip G3, other phyla similarly overrepresented on the PhyloChip G3 were not detected at comparable levels in the aeration-basin samples (Table S2).

### Common OTUs among the seasonal aeration-basin bacterial communities

Although the major phyla found in the seasonal samples were very similar, the samples shared a relatively low number of OTUs. A total of 1,027 OTUs (35% of the total OTUs counted, 33% of total species, 36% of total genus, 41% of total family, 44% of total order, 48% of total class and 52% of total phylum) were commonly detected in all field samples (Fig. 2). The common OTUs consisted of the phyla *Proteobacteria* (59%), *Bacteroidetes* (22%), *Firmicutes*, (11%), *Actinobacteria* (7%), and others (2%; *Planctomycetes*, *Gemmatimonadetes*, *Chloroflexi*, *Verrucomicrobia*, *Synergistetes*, *TM7*, *Acidobacteria*, *Tenericutes*, *GN04*, and *OP11*).

The common *Proteobacteria* consisted of the classes *β-Proteobacteria* (61%), *γ-Proteobacteria* (28%), *α-Proteobacteria* (10%), and *δ-Proteobacteria* (0.7%). In the *Bacteroidetes* phylum, the major classes were *Flavobacteria*, *Sphingobacteria*, and *Bacteroidia*. In the Firmicutes phylum, 67% was of the class *Clostridia*, and the rest was *Bacilli*. All of the common *Actinobacteria* OTUs belong to the class *Actinobacteria*.

### OTU variations by aeration-basin seasonal sampling dates

The bacterial communities collected at various sampling dates were distinguished from each other at hierarchical cluster level 3 based on the relative abundance of OTUs (Fig. S1). The differences among the bacterial communities by sampling dates based on the relative abundance of OTUs were moderate (MRPP: *A* = 0.47, *p* = 0.0004).

Indicator analysis was used to identify OTUs whose relative abundances significantly differed between sampling dates. The top 200 indicator OTUs (INDVAL≥40.5, *p*<0.05) are shown in Fig. S2. The indicator OTUs consisted of both major and minor classes. Among the 200 indicators, 98 OTUs were the indicators for the July 20, 2011 community, which belong to the classes *Actinobacteria*, *α-Proteobacteria*, *γ-Proteobacteria*, *Clostridia*, *Sphingobacteria*, *Planctomycea*, *β-Proteobacteria*, *Synergistia*, *Acidobacteria*, *δ-Proteobacteria*, *Erysipelotrichi*, *Solibacteres*, and unclassified *ABY1_OD1*. The October 19, 2011 community had 50 indicator OTUs, which belong to the classes *γ-Proteobacteria*, *β-Proteobacteria*, *Holophagae*, *Acidobacteria*, *Bacteroidia*, *Flavobacteria*, *α-Proteobacteria*, *Armatimonadia*, *Planctomycea*, and *Sphingobacteria*. The 52 indicator OTUs for the April 25, 2012 community belong to the classes *β-Proteobacteria*, *γ-Proteobacteria*, *Bacilli*, *Sphingobacteria*, *α-Proteobacteria*, *Clostridia*, and *ε-Proteobacteria*.

Taken together, the above results importantly establish a baseline for the seasonal variation of aeration-basin community composition.

### Laboratory culturing from aeration-basin inoculum

Flask-culture growth curves are presented in Figs. S3A and B. Growth with glucose had approximately a 10 h lag phase, and a peak OD_600 nm_ value of 0.5 was reached 21 h after inoculation. Growth with D-threonine and chloroacetate commenced following a 45 to 75-h lag phase. The standard deviations for all the replicates are small, except for the 50 mM chloroacetate condition, which attained the highest OD_600 nm_ (0.8) and contained 4.5 times more initial carbon than all other carbon sources. For both the [C2mim]Cl and no-carbon conditions, the OD_600 nm_ values did not change significantly, suggesting that [C2mim]Cl was tolerated but likely not metabolized. These results are important because they demonstrate that in addition to glucose, D-threonine and chloroacetate (up to 50 mM) can be effectively utilized as carbon sources by some of the aeration-basin microbes, although D-threonine and chloroacetate were associated with significantly longer lag phases than that observed for glucose. These results also show that no significant growth was observed in the [C2mim]Cl flask cultures despite monitoring three replicates through 140 h post inoculation.

Growth curves for the reactor-cultures are shown in Fig. S3C for glucose, D-threonine, and 11 and 23 mM chloroacetate. Despite irregularities in the curves due to air-bubble entrainment in the recycle line, and on some occasions due to adhesion of biomass, trends are consistent among replicates and these curves show more detail than the flask growth curves derived from intermittent sampling. For glucose, a 10 h lag period was followed by growth, which peaked at 20 h, similar to the flask-cultures. In the case of D-threonine, OD_600 nm_ values declined over the first 20 h, then increased beginning at 40 to 50 h, peaking at around 80 h, followed by a sharp decline. The flask culture did not show the sharp decline, although the peak occurred at around 70 h. 11 mM chloroacetate growth had two plateaus in succession, however the timing between the replicates varied; PhyloChip samples were taken after the second plateau. The replicates of the 23 mM chloroacetate runs were much more consistent than for 11 mM chloroacetate, each showing a 30 h lag, followed by growth to a plateau at 40 h. The flask-culture with 23 mM chloroacetate had much longer lag and growth phases compared to the reactor-cultures. In the no-carbon reactors (data not shown), OD_600 nm_ values dropped by 50% from their initial values in first 20 to 50 h, and remained constant thereafter.

### Composition of the laboratory-cultured bacterial communities

**Figure 3:**
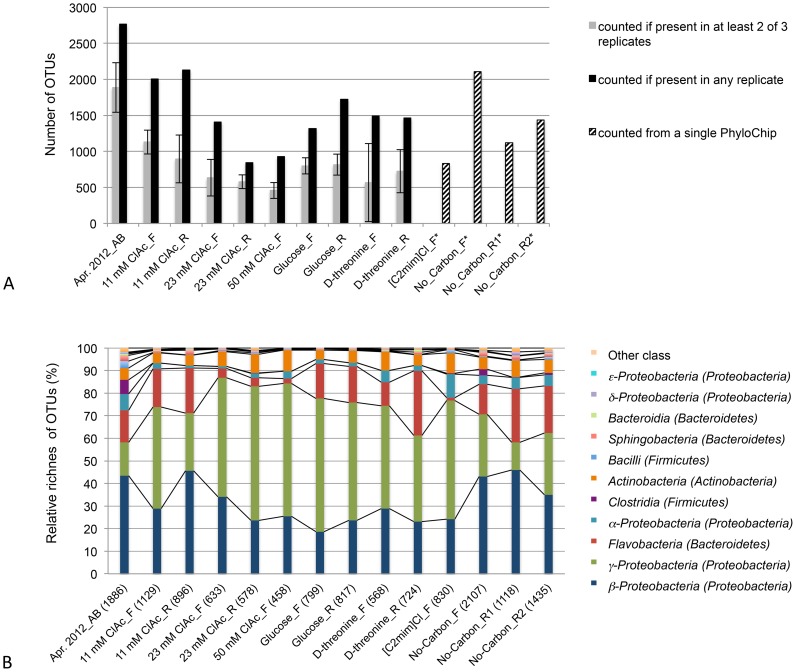
Number of OTUs and the class composition of the April, 2012 aeration-basin and laboratory-cultured samples. (A) Numbers of OTUs counted present in the preserved and maintained aeration-basin samples and the laboratory-cultured samples, as a function of the counting method. (B) Relative OTU richness at the class level. Relative OTU richness was calculated by dividing the number of OTUs (counted if present in at least two of three replicates) for a given class by the total number of OTUs in a given sample (shown in parentheses). _AB indicates aeration basin sample, _F indicates flask cultures, and _R indicates reactor cultures. An OTU was counted present if it was detected in at least two of three sample replicates, except for the [C2mim]Cl and No-Carbon flask-culture samples (three replicates were pooled together) and for the No_Carbon_R1 and No_Carbon_R2 (data from a single PhyloChip) (see Methods and Results).

The number of OTUs in each laboratory culture was reduced compared to its inoculum (the preserved and maintained April 25, 2012 aeration-basin sample; Fig. 3A, Table S2). Interestingly, large numbers of OTUs were detected in the [C2mim]Cl, and no-carbon cultures despite their very low cell mass. This is possibly a consequence of the less stringent positive OTU criteria used for these low cell mass bacterial communities (see Materials and methods). Briefly, for the low cell mass cultures, three replicates were pooled together and analyzed using a single PhyloChip, with any detected OTUs considered positives. In contrast, for the higher cell mass cultures, the three replicates were separately analyzed and positive OTUs were more stringently constrained to those detected in at least two replicates (Fig. 3A). The smallest number of OTUs (458) was detected in the 50 mM chloroacetate-cultured community, for which the largest OD_600 nm_ was measured.

The most abundant phyla in the laboratory-cultured communities were *Proteobacteria*, *Bacteroidetes*, and *Actinobacteria*. The *γ-Proteobacteria* was greatly enriched in almost all laboratory-cultured communities (Fig. 3B). While the *γ-Proteobacteria* made up only 15% of the total OTUs in the April 25, 2012 field community, it represented 45% of the total OTUs in the 11 mM chloroacetate, 59% in the 50 mM chloroacetate, 59% in the glucose, 46% in the D-threonine, and 53% in the [C2mim]Cl flask-cultured communities. In contrast, *β-Proteobacteria* was reduced in most laboratory-cultured communities. The *Clostridia* and *Bacilli* of the *Firmicutes* phylum were significantly reduced in all laboratory-cultured communities. The phylum *Bacteroidetes*, especially the *Flavobacteria* class, was greatly reduced in the communities cultured with chloroacetate at high concentrations, or with [C2mim]Cl. The *Actinobacteria* class was detected in all laboratory-cultured communities.

The result shown in Fig. 3 is important because it demonstrates that laboratory flasks and reactors-cultured diverse microbial communities (although the observed OTU richness was lower than that for the sampled aeration-basin community) and specific classes were differentially affected by both the culturing method and selective media components.

### Similarities among the aeration-basin field communities and the laboratory-cultured communities

**Figure 4:**
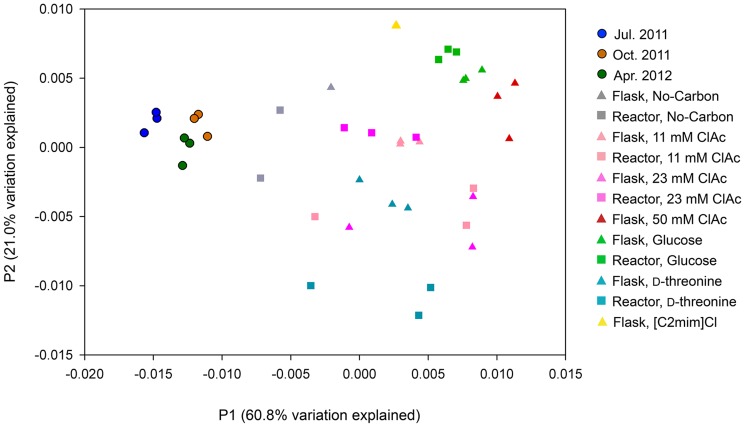
Principal Coordinate Analysis (PCoA) of the aeration-basin and the laboratory-cultured sample bacterial communities. Samples are projected onto the first (P1) and second (P2) principal coordinate axes. Three sample replicates are plotted for each community, except for the [C2mim]Cl and No-Carbon flask laboratory-culture samples, for which the three replicates were pooled together (see Methods and Results).

Microbial composition similarities among the communities were inferred by PCoA, which employed UniFrac distance measurement (Fig. 4). The aeration-basin communities were differentiated from the laboratory-cultured communities along the P1 axis, which explained the majority of total variation (60.8%). The P2 (21.0% of total variation) and P3 (6.8% of total variation; Fig. S4) axes further differentiated the laboratory-cultured communities.

**Figure 5:**
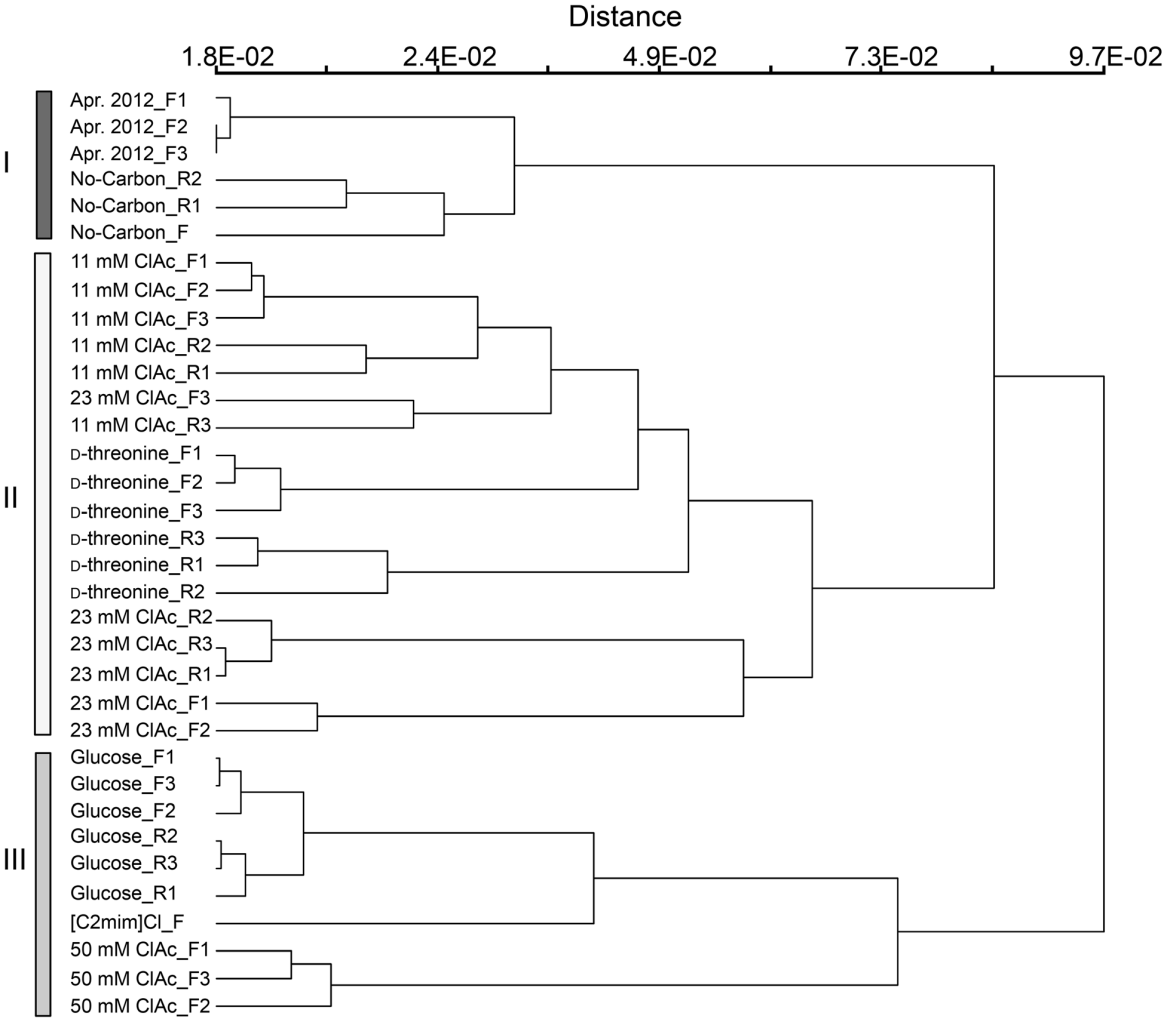
Hierarchical clustering of the aeration-basin and the laboratory-cultured samples. The clustering was constructed with the relative OTU abundance data employing the Bray-Curtis distance measurement and group average linkage methods. _F indicates flask cultures and _R indicates reactor cultures.

Hierarchical cluster analysis using Bray-Curtis distance matrix and group average linkage method supported the PCoA analysis result (Fig. 5). The April 25, 2012 field communities and no-carbon-cultured communities were grouped together (cluster I) and were distinct from the laboratory-cultured communities. Cluster II, which consisted of the 11 mM and 23 mM chloroacetate and the D-threonine-cultured communities, was more closely located to cluster I containing the April 25, 2012 communities compared to cluster III, which contained the glucose, [C2mim]Cl, and 50 mM chloroacetate-cultured communities.

### Comparison of the microbial richness among the April 25, 2012 aeration-basin community and the laboratory-cultured communities

**Figure 6:**
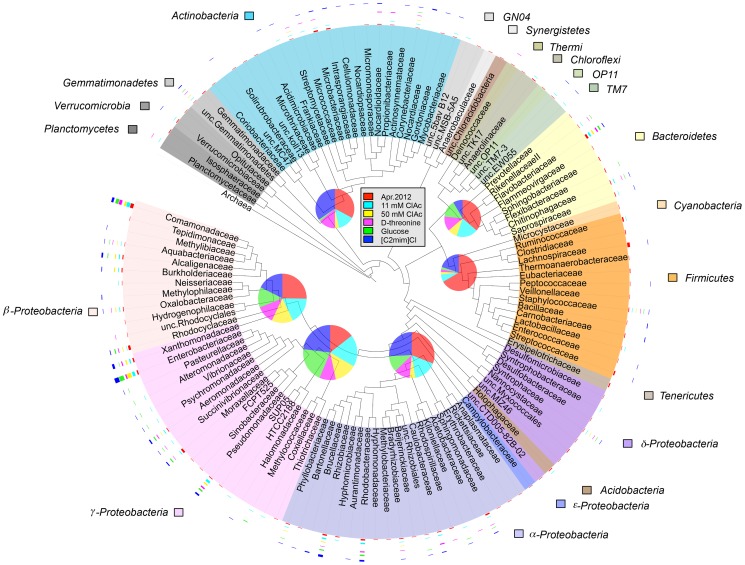
Microbial composition of the April 25, 2012 aeration-basin and flask-cultured samples. FastTree was used to generate an approximate-maximum-likelihood phylogenetic tree, which was rooted with archaea. The leaves of the tree list taxa at the family level, or for unclassified (unc.) families the lowest ancestor. Families belonging to the same phylum (or, class level for the phylum *Proteobacteria*) are shaded in the same color. Band intensity graphs outside each leaf (family) indicate the number of species in the family detected in each sample. Pie charts at the nodes of major phyla (or, classes) show the number of species in each sample. Pie chart area correlates with the total number of the species of the given phylum, or class. The pie and band intensity graphs do not share the same scale (the minimum number of species in each band intensity graph is 1, and the maximum is 15; in each pie chart, the minimum number of species is 1, and the maximum is 38). An OTU was counted present if it was detected in at least two of three sample replicates, except for the [C2mim]Cl samples, for which the three replicates were pooled together (see Methods and Results).

A total of 120 families were found in the April 25, 2012 samples and the flask-cultured communities (Fig. 6). There were 83 families in the April 25, 2012 field community, 49 families in 11 mM chloroacetate, 20 families in 50 mM chloroacetate, 31 families in D-threonine, 29 families in glucose, and 56 families in [C2mim]Cl cultures. The families detected in the laboratory cultures were phylogenetically diverse as shown in the tree in Fig. 6. In general, the April 25, 2012 sample contained more diverse species and families in most phyla. The following families in particular contained more diverse species in the aeration-basin community: *Lachnospiraceae, Ruminococcaceae*, *Streptococcaceae*, *Clostridiaceae*, and *Eubacteriaceae* (belonging to the *Firmicutes* phylum), *Rhodocyclaceae* (belonging to the *β-Proteobacteria* class), *Moraxellaceae* (*γ -Proteobacteria*), *Gemmatimonadaceae* (*Gemmatimonadetes*), *Isosphaeraceae* (*Planctomycetes*), and *Sphingobacteriaceae* (*Bacteroidetes*). In contrast, the *γ-Proteobacteria* were more diverse in some of the flask-cultured communities than in the aeration-basin community: April 25, 2012 community (24 species), glucose (34 species), [C2mim]Cl (38 species), 11 mM chloroacetate (31 species), 50 mM chloroacetate (22 species) and D-threonine (16 species). The *Actinobacteria* phylum contained a comparable number of species between the [C2mim]Cl (20 species) and the field community (19 species).

### Comparison of the microbial abundance in the laboratory-cultured communities

The relative abundances of the top 50 families are shown in Fig. S5. Most families showed stronger microarray intensities in the aeration-basin communities than in the laboratory-cultured communities. However, *Enterobacteriaceae*, *Aeromonadaceae* and *Succinivibrionaceae* belonging to the *γ-Proteobacteria* class were more relatively abundant in the glucose and [C2mim]Cl-cultured communities. It appears that [C2mim]Cl also enriched *Xanthomonadaceae* (*γ-Proteobacteria*), *Rhizobiaceae* (*α-Proteobacteria*), and *Micrococcaceae* (*Actinobacteria*). The *Pseudomonadaceae* (*γ-Proteobacteria*) was enriched the most in the 23 mM chloroacetate reactor cultures, while *Oxalobacteraceae* (*β-Proteobacteria*) was enriched the most in the 23 mM chloroacetate flask cultures. The *Burkholderiaceae* (*β-Proteobacteria*) was most abundant in the 50 mM chloroacetate cultures.

**Figure 7:**
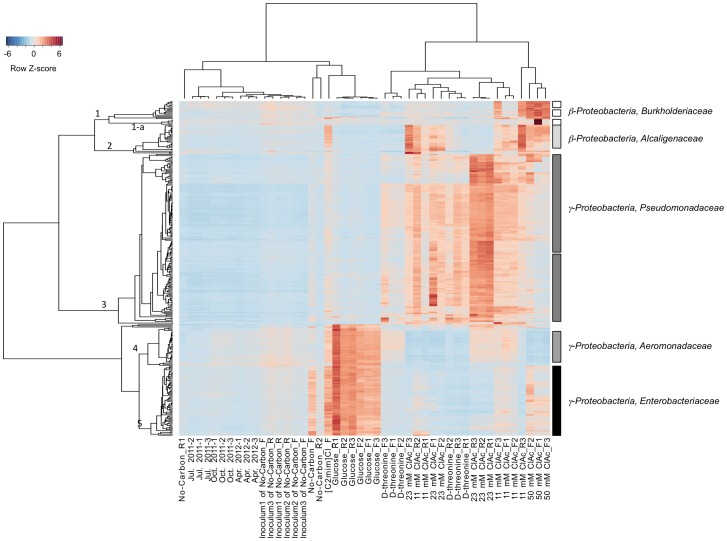
OTUs enriched by chloroacetate, glucose, or D-threonine. Microarray relative intensities for the OTU indicators (rows) are shown across samples (columns). The color gradient from blue to red represents increasing microarray intensity.

Significantly enriched individual OTUs were identified by hierarchical clustering using a Bray-Curtis distance matrix and the flexible beta linkage method followed by indicator analysis. OTUs (minimum INDVAL≥66.7, *p*<0.05) enriched in laboratory-cultured communities are shown together in Fig. 7. The majority of enriched microbes were *γ-Proteobacteria*.

Ninety-three percent of the top 100 enriched OTUs in the glucose and [C2mim]Cl -cultured communities belong to *Enterobacteriaceae* (the genus *Cronobacter*, *Erwinia*, *Escherichia*, *Klebsiella*, *Pantoea*, *Raoultella*, *Salmonella*, *Serratia*, *Sodalis*, and unclassified) and *Aeromonadaceae* families (cluster 5 and cluster 4, Fig. 7). Interestingly, these *Enterobacteriacea* OTUs appeared to be enriched in the no-carbon added flask culture as well, but not in the no-carbon added reactor cultures. In contrast, the *Aeromonadaceae* OTUs were not enriched in any of no-carbon added cultures.

Ninety-eight percent of the enriched OTUs by D-threonine were *Pseudomonadaceae* (cluster 3, Fig. 7). Most of them were unclassified *Pseudomonas* species and the rests were classified *Pseudomonas* species (*P. fragi*, *P. fuscovaginae*, *P. lutea*, *P. mendocina*, *P. mosselii, P. nitroreducens*, *P. stutzeri*, *P. veronii*, and *P. viridiflava*), unclassified *δ-Proteobacteria*, and *Flavobacteriaceae*.

In all 11 mM chloroacetate and 23 mM chloroacetate flask cultures, the majority of the indicator OTUs were *Pseudomonadaceae* (*P. fuscovaginae*, *P. mendocina*, *P. nitroreducens*, *P. stutzeri*, *P. veronii* and unclassified *Pseudomonas* sp.) and *Alcaligenacea* (*β-Proteobacteria*) (cluster 2, Fig. 7). The rests were *Oxalobacteraceae* (*β-Proteobacteria*), unclassified *δ-Proteobacteria* and *Flavobacteriaceae*.

The *Pseudomonadaceae* family was also enriched in the 23 mM chloroacetate reactor culture. However, no indicator OTU belonged to the *Alcaligenaceae* family, unlike all other chloroacetate-cultured communities.

The high concentration (50 mM) of chloroacetate enriched various *β-Proteobacteria* (the families *Burkholderiaceae*, *Alcaligenaceae*, *Oxalobacteraceae*, and *Methylibiaceae*), as well as *Pseudomonadaceae*. The enrichment of *Burkholderiaceae* was characteristic for this community. The *Burkholderiaceae* OTU indicators consisted genus *Burkholderia* (18 OTUs) and *Ralstonia* (2 OTUs). Some of the *Burkholderia* OTUs in one of the replicate cultures were extremely enriched showing approximately 10–60 times stronger microarray intensities (cluster 1-a, Fig. 7).

The results shown in Figs. 7 and S5 demonstrate that glucose, D-threonine, chloroacetate, and [C2mim]Cl differentially affect community composition, a key characteristic of selective media. Furthermore, these results show specifically which subsets of the community are respectively enriched in the presence of glucose, D-threonine, chloroacetate, or [C2mim]Cl, establishing baselines for aeration-basin community composition changes following propagation in laboratory flask or reactor culture.

## Discussion

We report here our foundational efforts towards the development of a laboratory model of an industrial bioreactor failure scenario that introduces genetically engineered microorganisms (GEMs) to a downstream municipal wastewater treatment plant (MWWTP). Our first step was to sample microbial communities from the MWWTP's aeration basin (a potential hot spot for horizontal gene transfer) at three seasonal time points to establish a baseline for a MWWTP community composition (Fig. 2). Since microbial community composition in activated sludge treatment systems is dynamic and can be affected by various environmental factors including temperature and sources of wastewater influx, it is not surprising that we observed moderate variation in composition between our July 20, 2011, October 19, 2011, and April 25, 2012 aeration-basin samples. Although the number of OTUs detected in common across all samples was relatively low, the microbial composition at the higher taxa level was very similar (Fig. 2). It is noteworthy, though, that the OTUs of the PhyloChip G3 are at the very fine taxonomic level (sub-species) and have very low sequence divergence among them (an average of 0.5%) [19]. The major phyla of the aeration-basin community that we found (*Proteobacteria*, *Bacteroidetes*, *Firmicutes*, and *Actinobacteria*) are similar to those found in a previous study of the microbial community of a Hong Kong wastewater plant that employed DNA and cDNA sequence analysis [8]. In contrast to this previous study that examined only one seasonal community at the phylum level (except selectively examining the microbes involved in nitrification at the genus level), our work investigated microbial communities at various taxonomic levels in depth and also compared the communities between seasons. Our observations place weight on the importance of pursuing multi-seasonal assessment of GEM survival and gene transfer rates within MWWTP microbial communities in future research efforts. At the same time, the moderate extent of seasonal variation in microbial community composition provides a degree of confidence that an assessment made at a particular seasonal time point will be representative of other time points.

Having established a baseline for seasonal variation in aeration-basin community composition, we then investigated how the community changed when propagated in the laboratory. Laboratory flask and reactor systems cultured fairly similar yet distinct microbial communities (Figs. 3–5, 7, S3–5). While a proper bench-scale simulation of an activated sludge treatment system requires the use of a bioreactor, the observed similarity supports the utility of screening carbon sources and growth conditions in flasks before progressing to reactors. While laboratory-cultured communities generally contained fewer taxa, the major phyla and classes present in the aeration-basin communities were also detected in the laboratory-cultured communities, which retained reasonably high phylogenetic diversity. Some OTUs, especially belonging to *Firmicutes* that was one of the major phyla in the MWWTP communities, were much less abundant in laboratory cultures than in aeration-basin communities. We speculate that differences in oxygen concentrations and distributions (and the dynamics thereof) between small-scale laboratory flask and reactor cultures and the large-scale wastewater treatment plant could explain some of the observed composition differences, such as the decreased abundance of anaerobic *Clostridia* in laboratory cultures (Fig. 3B). While the mixed liquor is intensively aerated and mixed in the wastewater treatment plant aeration basin, the passage of the mixed liquor through a non-aerated clarifier could transiently support the growth of *Clostridia*. There is no equivalent passage through a non-aerated compartment in the laboratory culture system, which is constantly aerated to saturation. In contrast, some OTUs were more abundant (or, detected only) in laboratory-cultured communities. The various laboratory culture conditions may have enriched for microbes otherwise in low-abundance in the aeration-basin.

Beyond assessing the extent to which our laboratory growth conditions simulated those of the aeration-basin, we tested the effectiveness of chloroacetate and D-threonine carbon sources and the microbial growth inhibitor [C2mim]Cl as selective media component candidates for future GEM survival and gene transfer experiments. We anticipated that these media amendments would promote the activity of carbon catabolic or efflux-pump tolerance genes, rare in MWWTP microbial communities, which could serve as selective markers in these future studies. Such a future study would introduce a GEM (containing a carbon catabolic or efflux-pump tolerance selectable marker) to our aeration-basin laboratory model under the appropriate selective growth condition, and then monitor the survival of the GEM and the horizontal transfer and propagation of the portion of its DNA encoding the marker.

Although the 50 mM chloroacetate flask cultures attained the highest OD_600 nm_ (Fig. S3), they contained the smallest number of OTUs (485) of all the conditions. This suggests that the diversity of microbes that can survive in high concentration of chloroacetate is relatively low. The genus *Burkholderia*, in particular, was enriched in the 50 mM chloroacetate flask cultures (Figs. 7, S5). The haloacetate dehalogenase (EC.3.8.1.3) from a *Burkholderia* species had been characterized [32] to catabolize chloroacetate to glycolate. Glycolate, in turn, can be oxidized to glyoxylate by glycolate oxidase and used as a carbon and energy source. The genomic information of *B. gladioli*, *B. mallei*, and *B. phytofirmans* available at MetaCyc (Stanford Research Institute International [http://metacyc.org]) [33] indicates that these three species have genes encoding haloacid dehalogenase (EC.3.8.1.2). To this end, the unclassified *Burkholderia* species detected in this study may be similarly able to metabolize chloroacetate. The *Pseudomonas* genus was enriched by chloroacetate at all concentrations tested (11, 23, and 50 mM; Fig. 7). The haloacid dehalogenase of *Pseudomonas* sp. YL and *P. putida* had been characterized, and an *E. coli* overexpressing haloacid dehalogenase can metabolize chloroacetate as a sole energy source [34]–[39]. While *Pseudomonas* sp. YL and *P. putida* were not detected in this study, the unclassified *Pseudomonas* species found in this work may have a similar ability to catabolize chloroacetate. The *Alcaligenaceae* family (*Achromobacter* genus, unclassified species) was also enriched by chloroacetate (Fig. 7). Although not detected in this work, *A*. *xylosoxidans*, a species belonging to *Achromobacter*, has a gene encoding haloacetate dehalogenase. Taken together, these three observations support chloroactetate as a selective media component candidate, and haloacid dehalogenase as a selective marker, for our future GEM survival and gene transfer experiments: 1) *Burkholderia*, *Pseudomonas*, and *Achromobacter* were all significantly enriched in the chloroacetate-amended laboratory cultures; 2) in these same genera, haloacid dehalogenases have previously been identified, heterologously expressed and characterized; and 3) haloacid dehalogenase can not only detoxify chloroactetate to glycolate, but also enable the metabolism of chloroacetate as a sole energy source.

Beyond chloroacetate, many of the *Pseudomonas* and some of the *Alcaligenaceae* were also enriched by D-threonine. D-threonine aldolase catabolizes D-threonine into glycine and acetaldehyde, and acetaldehyde feeds into the TCA cycle. The D-threonine aldolase of *Pseudomonas* has not been studied in depth, but the enzyme was characterized in *Xanthomonadaceae* (*Xanthomonas oryzae*) and *Alcaligenaceae* (*Achromobacter xylosoxidans*), which are closely related to the OTUs detected in the D-threonine cultures [40], [41]. These observations support D-threonine as an additional selective media component candidate, and D-threonine aldolase as an additional selective marker, for our future experiments.

Few microbes have been shown to grow in the presence of the ionic liquid [C2mim]Cl [14]. Although our OD_600 nm_ measurements did not detect any net growth in the presence of [C2mim]Cl (Fig. S3B), a high number of OTUs was detected for the [C2mim]Cl sample. We suspect that although many microbes may have been non-viable, dead, or dormant at the time of [C2mim]Cl culture sampling, their respective DNA persisted in the medium long enough for PhyloChip detection. To distinguish which OTUs are active, in future experiments, expressed rRNA could be applied to the PhyloChip and recovery experiments and live/dead staining could be performed. Given the relatively high abundance in the [C2mim]Cl sample of the *Xanthomonadaceae*, *Rhizobiaceae*, *Micrococcaceae*, *Enterobacteriaceae*, *Aeromonadaceae*, and *Alcaligenaceae* families (Figs. S5, 7), it appears that microbes belonging to these families may have persisted or propagated (under the OD_600_ detection limit) better than others in the presence [C2mim]Cl. Since an efflux pump for [C2mim]Cl tolerance was identified in *Enterobacter lignolyticus* SCF1 and cross-validated in *E. coli* [14], [C2mim]Cl may also prove to be a valuable selective media component candidate in our future experiments.

Here, we demonstrated the capacity to culture a microbial community using preserved inocula from a municipal wastewater treatment plant, and maintain adequate diversity and repeatability to test GEM survival and gene transfer rates in laboratory-scale model system. Our results show specifically which subsets of the community are enriched in the presence of glucose, D-threonine, chloroacetate, or [C2mim]Cl, establishing baselines for aeration-basin community composition changes following propagation in laboratory flask or reactor culture. The observation that microbial communities cultured with 11 or 23 mM chloroacetate and D-threonine are more similar to the sampled aeration-basin communities than those cultured with glucose, [C2mim]Cl, or 50 mM chloroacetate (Fig. 5), may prove useful when choosing a particular selective medium over another. Our analysis supports haloacid dehalogenase, D-threonine aldolase, and the [C2mim]Cl efflux pump from *Enterobacter lignolyticus* SCF1 as candidate selective markers, and chloroactetate, D-threonine, and [C2mim]Cl as selective media components, respectively, for our future GEM survival and gene transfer experiments. This work establishes a methodological foundation for assessing and mitigating the risks of future large-scale microbial metabolic engineering projects, including those extending beyond the bioreactor.

## Supporting Information

Figure S1 **Hierarchical clustering of the seasonal aeration-basin samples based on the relative OTU abundances.** The Bray-Curtis distance measurement and group average linkage methods were employed for the clustering.
(TIF)

Figure S2 **Indicator analysis of the seasonal aeration-basin sample relative OTU abundances.** Microarray relative intensities for the top 200 OTU indicators (rows; indicator value >40.5, *p*<0.05) are shown for each sample replicate (columns). The color gradient from blue to red represents increasing microarray intensity.
(TIF)

Figure S3 **Microbial growth curves.** (A, B) Flask culture growth curves for (A) glucose, D-threonine, chloroacetate carbon sources; and (B) [C2mim]Cl and no carbon source. Curves are the average of triplicates with error bars representing one standard deviation between replicates. (C) Reactor culture growth curves (three replicate curves each) for glucose, D-threonine, chloroacetate carbon sources. Optical density was measured continuously by means of spectrophotometer in recycle line of reactor, with oscillations resulting from air-bubble entrainment in the recycle line or other irregularities due to the adhesion of biomass.
(TIF)

Figure S4 **Principal Coordinate Analysis (PCoA) of the aeration-basin and the laboratory-cultured sample bacterial communities.** Samples are projected onto the first (P1) and third (P3) principal coordinate axes. Three sample replicates are plotted, except for the [C2mim]Cl and No-Carbon laboratory-culture samples, for which the three replicates were pooled together (see Methods and Results).
(TIF)

Figure S5 **Comparison of the relative family abundances among the aeration-basin and the laboratory-cultured samples.** Families ranking within the top 50 of the April 2012 sample are shown. The color gradient from blue to red represents increasing microarray intensity. The intensity of each family was calculated by adding all the intensity values of the OTUs belonging to that family. Each row represents a family and each column represents a sample.
(TIF)

Table S1 **The NCBI-ID of the representative OTUs for each family used in the phylogenetic tree in the** **Fig. 6** **.**
(DOC)

Table S2 **The number of OTUs comprising each phylum.**
(DOC)
