# Supplementary figures and images for: Characterization of Wastewater Treatment Plant Microbial Communities and the Effects of Carbon Sources on Diversity in Laboratory Models
Source: PLoS One. 2014 Aug 22;9(8):e105689. doi: 10.1371/journal.pone.0105689 (PMC4141834; doi:10.1371/journal.pone.0105689)

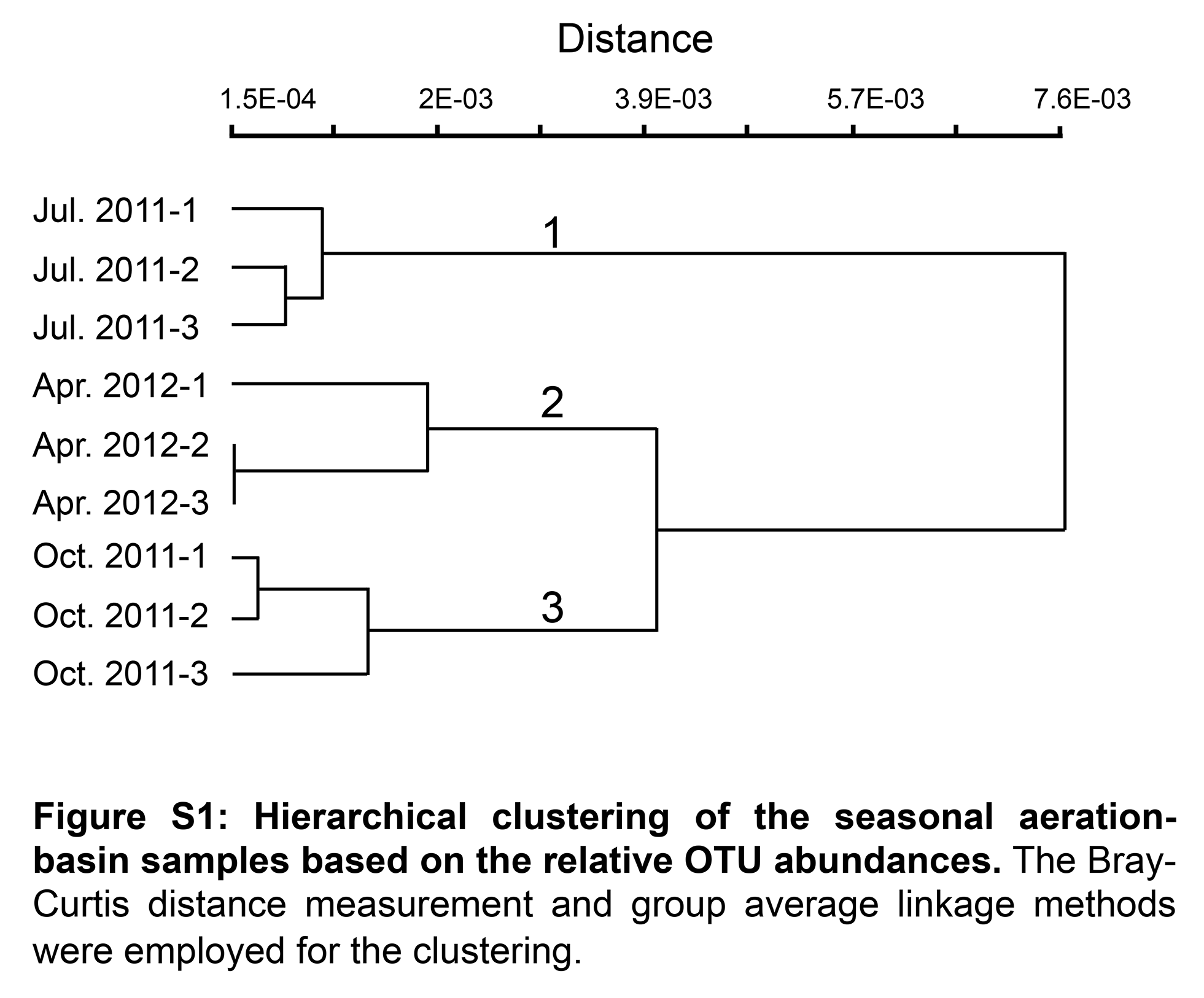

Supplement: Figure S1 — Hierarchical clustering of the seasonal aeration-basin samples based on the relative OTU abundances. The Bray-Curtis distance measurement and group average linkage methods were employed for the clustering. (TIF) [file pone.0105689.s001.tif]

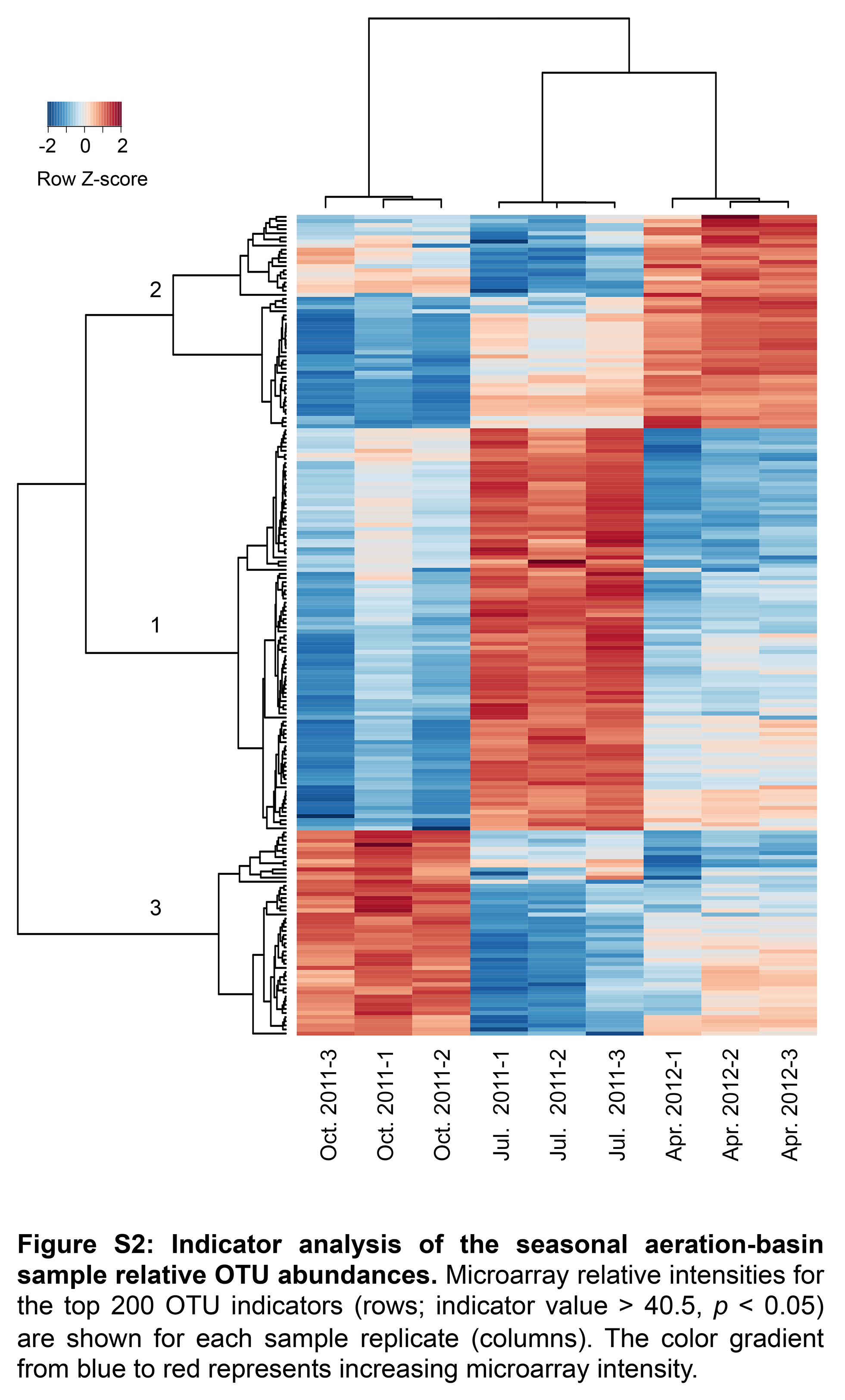

Supplement: Figure S2 — Indicator analysis of the seasonal aeration-basin sample relative OTU abundances. Microarray relative intensities for the top 200 OTU indicators (rows; indicator value >40.5, p<0.05) are shown for each sample replicate (columns). The color gradient from blue to red represents increasing microarray intensity. (TIF) [file pone.0105689.s002.tif]

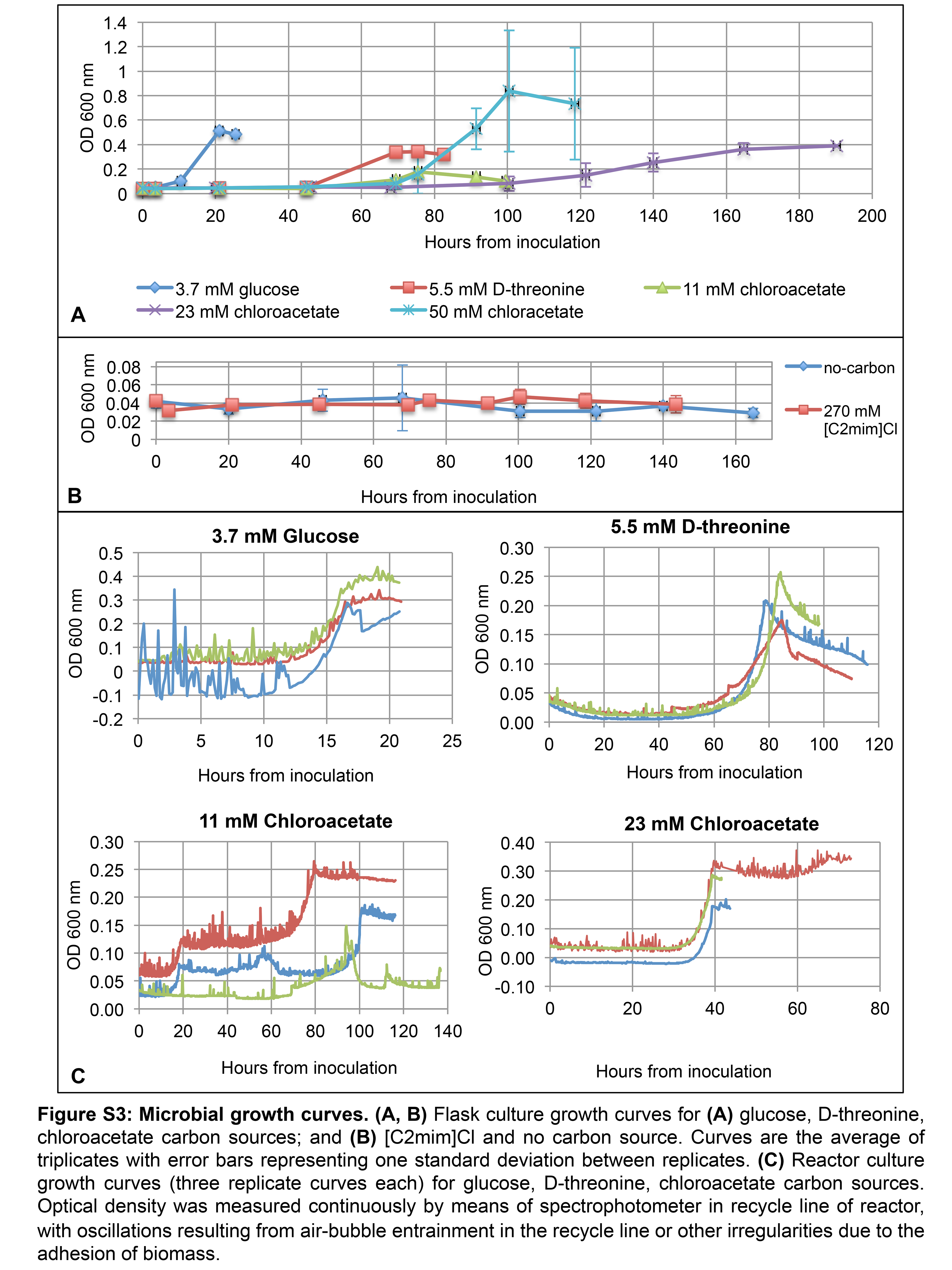

Supplement: Figure S3 — Microbial growth curves. (A, B) Flask culture growth curves for (A) glucose, D-threonine, chloroacetate carbon sources; and (B) [C2mim]Cl and no carbon source. Curves are the average of triplicates with error bars representing one standard deviation between replicates. (C) Reactor culture growth curves (three replicate curves each) for glucose, D-threonine, chloroacetate carbon sources. Optical density was measured continuously by means of spectrophotometer in recycle line of reactor, with oscillations resulting from air-bubble entrainment in the recycle line or other irregularities due to the adhesion of biomass. (TIF) [file pone.0105689.s003.tif]

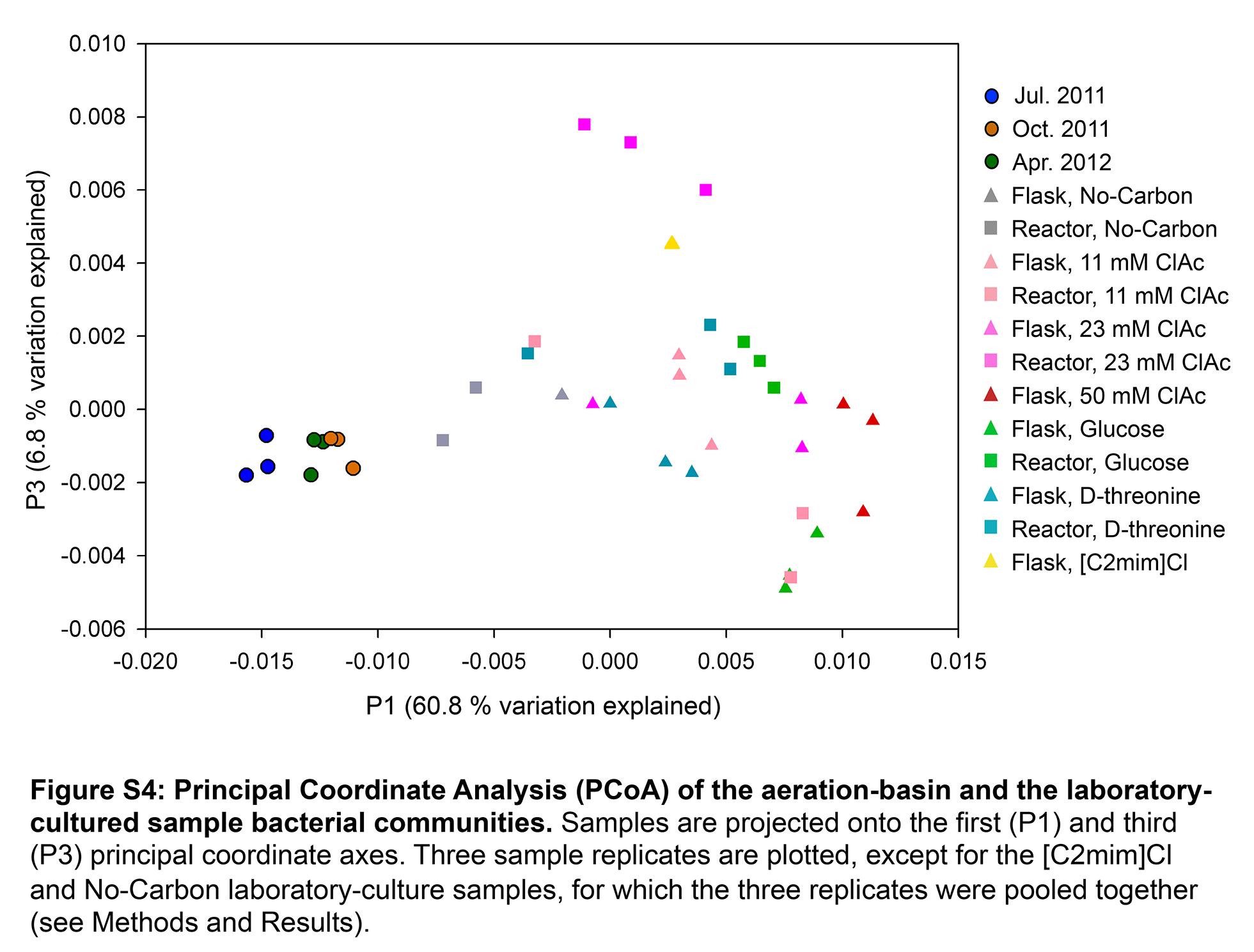

Supplement: Figure S4 — Principal Coordinate Analysis (PCoA) of the aeration-basin and the laboratory-cultured sample bacterial communities. Samples are projected onto the first (P1) and third (P3) principal coordinate axes. Three sample replicates are plotted, except for the [C2mim]Cl and No-Carbon laboratory-culture samples, for which the three replicates were pooled together (see Methods and Results). (TIF) [file pone.0105689.s004.tif]

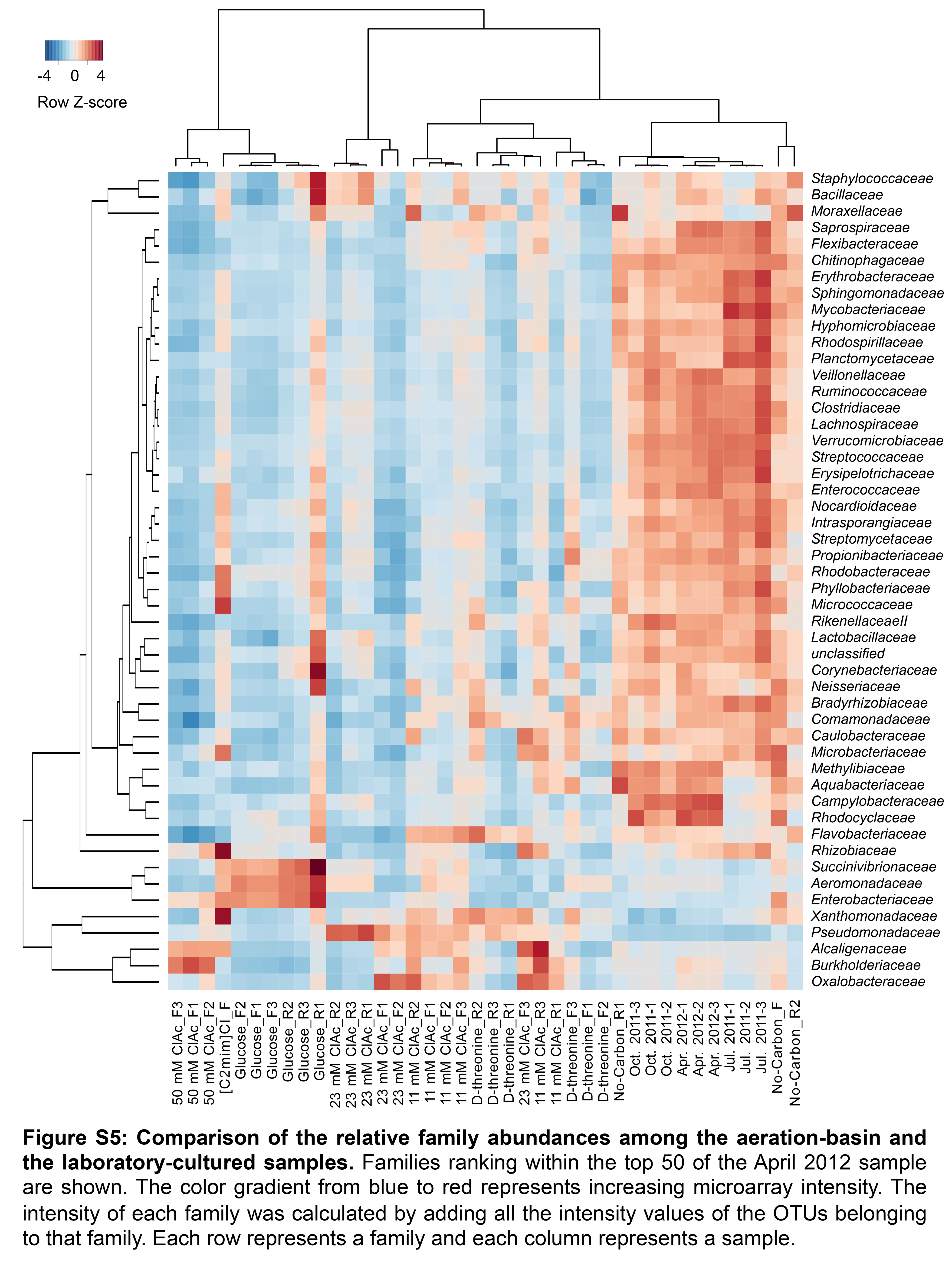

Supplement: Figure S5 — Comparison of the relative family abundances among the aeration-basin and the laboratory-cultured samples. Families ranking within the top 50 of the April 2012 sample are shown. The color gradient from blue to red represents increasing microarray intensity. The intensity of each family was calculated by adding all the intensity values of the OTUs belonging to that family. Each row represents a family and each column represents a sample. (TIF) [file pone.0105689.s005.tif]
