# Supplementary material for: Characterization of Wastewater Treatment Plant Microbial Communities and the Effects of Carbon Sources on Diversity in Laboratory Models
Source: PLoS One. 2014 Aug 22;9(8):e105689. doi: 10.1371/journal.pone.0105689 (PMC4141834; doi:10.1371/journal.pone.0105689)
Supplement: Table S1 — The NCBI-ID of the representative OTUs for each family used in the phylogenetic tree in the Fig. 6 . (DOC) [file pone.0105689.s006.doc]

**Table S1: The NCBI-ID of the representative OTUs for each family used in the phylogenetic tree in the Fig. 6.**

| Family | NCBI ID for a representative OTU |  |  |  |  |  |  |
| --- | --- | --- | --- | --- | --- | --- | --- |
| *Acetobacteraceae* | EF019939.1 |  | *Gemmatimonadaceae* | EU652636.1 |  | *Rhodobacteraceae* | D32241.1 |
| *Acidimicrobiaceae* | FJ466121.1 |  | *Gordoniaceae* | AY650265.3 |  | *Rhodocyclaceae* | AF229868.1 |
| *Actinosynnemataceae* | EU570742.1 |  | *Halomonadaceae* | GU397429.1 |  | *Rhodospirillaceae* | AY563022.1 |
| *Aeromonadaceae* | AB211226.1 |  | *Holophagaceae* | EU803304.1 |  | *Rickettsiaceae* | U12458.1 |
| *Alcaligenaceae* | AB234302.1 |  | *HTCC2188* | EU799676.1 |  | *RikenellaceaeII* | AB504929.1 |
| *Alteromonadaceae* | AY820719.1 |  | *Hydrogenophilaceae* | GU208503.1 |  | *Ruminococcaceae* | EU887966.1 |
| *Anaerobaculaceae* | EU276415.1 |  | *Hyphomicrobiaceae* | FN421512.1 |  | *Saprospiraceae* | AM286229.1 |
| *Anaerolinaceae* | EU134013.1 |  | *Hyphomonadaceae* | AB286463.1 |  | *Sinobacteraceae* | GQ214101.1 |
| *Anaplasmataceae* | FJ478621.1 |  | *Intrasporangiaceae* | AF409018.1 |  | *Solirubrobacteraceae* | EU289476.1 |
| *Aquabacteriaceae* | GQ093131.1 |  | *Isosphaeraceae* | AB254788.1 |  | *Sphingobacteriaceae* | FN421509.1 |
| *Aurantimonadaceae* | EU544515.1 |  | *Kiloniellaceae* | AB373113.1 |  | *Sphingomonadaceae* | AB074191.1 |
| *Bacillaceae* | EF063149.1 |  | *Lachnospiraceae* | DQ795187.1 |  | *Staphylococcaceae* | EU434592.1 |
| *Bartonellaceae* | AY515118.1 |  | *Lactobacillaceae* | AY735404.1 |  | *Streptococcaceae* | AY762104.1 |
| *Beijerinckiaceae* | AJ563926.1 |  | *Methylibiaceae* | EU639232.1 |  | *Streptomycetaceae* | AM040290.1 |
| *Bradyrhizobiaceae* | AF288309.1 |  | *Methylobacteriaceae* | AY366072.1 |  | *Succinivibrionaceae* | FJ673291.1 |
| *Brucellaceae* | AJ550273.2 |  | *Methylococcaceae* | AF150807.1 |  | *SUP05* | AB278146.1 |
| *Burkholderiaceae* | AB025790.1 |  | *Methylophilaceae* | DQ513082.1 |  | *Syntrophaceae* | AF482437.1 |
| *Campylobacteraceae* | AJ607391.1 |  | *Microbacteriaceae* | DQ227776.1 |  | *Syntrophobacteraceae* | AM997396.1 |
| *Carnobacteriaceae* | AJ306612.1 |  | *Micrococcaceae* | AJ551149.1 |  | *Tepidimonaceae* | AF309815.1 |
| *Caulobacteraceae* | AJ495803.1 |  | *Microcystaceae* | D89031.1 |  | *Thermoanaerobacteraceae* | FJ189582.1 |
| *Cellulomonadaceae* | AJ292035.1 |  | *Micromonosporaceae* | EU132986.1 |  | *Thiotrichaceae* | AB042542.1 |
| *Chitinophagaceae* | AB470450.1 |  | *Microthrixaceae* | DQ450797.1 |  | *unc.5bav_B12* | GU061180.1 |
| *Clostridiaceae* | EU470820.1 |  | *Moraxellaceae* | AB176230.1 |  | *unc.Chloracidobacteria* | EU131934.1 |
| *Comamonadaceae* | GQ075548.1 |  | *Mycobacteriaceae* | AF480582.1 |  | *unc.CTD005-82B-02* | EU978839.1 |
| *Coriobacteriaceae* | EU766266.1 |  | *Nannocystaceae* | EU283398.1 |  | *unc.EW055* | AB200304.1 |
| *Corynebacteriaceae* | FJ675067.1 |  | *Neisseriaceae* | L06174.1 |  | *unc.Gemmatimonadetes* | EU979063.1 |
| *Coxiellaceae* | EU491737.1 |  | *Nocardiaceae* | AM746989.1 |  | *unc.koll13* | FJ229962.1 |
| *Deinococcaceae* | DQ683348.1 |  | *Nocardioidaceae* | CU922508.1 |  | *unc.MC47* | GU208243.1 |
| *Desulfobacteraceae* | AJ704696.1 |  | *Nocardiopsaceae* | AB006171.1 |  | *unc.MIZ46* | FJ542849.1 |
| *Desulfomicrobiaceae* | DQ069204.1 |  | *Opitutaceae* | AY297806.1 |  | *unc.MSB-5A5* | DQ811954.1 |
| *Enterobacteriaceae* | AY259630.1 |  | *Oxalobacteraceae* | AB512141.1 |  | *unc.Myxococcales* | AY922068.1 |
| *Enterococcaceae* | AJ301838.1 |  | *Pasteurellaceae* | EU185421.1 |  | *unc.OP11* | DQ298014.1 |
| *Erysipelotrichaceae* | AB030222.1 |  | *Peptococcaceae* | FJ671684.1 |  | *unc.Rhizobiales* | AY792286.1 |
| *Erythrobacteraceae* | DQ856560.1 |  | *Phyllobacteriaceae* | D32248.1 |  | *unc.Rhodocyclales* | AY212696.1 |
| *Eubacteriaceae* | AF132739.1 |  | *Planctomycetaceae* | AB486437.1 |  | *unc.TK17* | FJ746106.1 |
| *FCPT525* | EU104152.1 |  | *Prevotellaceae* | AJ581354.1 |  | *unc.TM7-3* | EU979068.1 |
| *Flammeovirgaceae* | EU328009.1 |  | *Propionibacteriaceae* | GQ091123.1 |  | *Veillonellaceae* | AB486202.1 |
| *Flavobacteriaceae* | AJ715377.1 |  | *Pseudomonadaceae* | AB001446.1 |  | *Verrucomicrobiaceae* | FJ437966.1 |
| *Flexibacteraceae* | DQ335125.1 |  | *Psychromonadaceae* | AJ519627.1 |  | *Vibrionaceae* | AB457055.1 |
| *Frankiaceae* | AJ408874.1 |  | *Rhizobiaceae* | AF417565.1 |  | *Xanthomonadaceae* | AJ519985.1 |
