# Supplementary material for: Characterization of Wastewater Treatment Plant Microbial Communities and the Effects of Carbon Sources on Diversity in Laboratory Models
Source: PLoS One. 2014 Aug 22;9(8):e105689. doi: 10.1371/journal.pone.0105689 (PMC4141834; doi:10.1371/journal.pone.0105689)
Supplement: Table S2 — The number of OTUs comprising each phylum. (DOC) [file pone.0105689.s007.doc]

**Table S2: The number of OTUs comprising each phylum^*^.**

|  | PhyloChi G3 | Jul. 2011 | Oct. 2011 | Apr. 2012 | 11 mM | 11 mM | 23 mM | | 23 mM | | 50 mM | | Glucose | Glucose | D-threonine | D-threonine | [C2mim]Cl | No-Carbon | No-Carbon | No-Carbon |
| --- | --- | --- | --- | --- | --- | --- | --- | --- | --- | --- | --- | --- | --- | --- | --- | --- | --- | --- | --- | --- |
|  |  | AB | AB | AB | ClAc_F | ClAc_R | ClAc_F | | ClAc_R | | ClAc_F | | _F | _R | _F | _R | _F | _F | _R1 | _R2 |
| ***ABY1_OD1*** | 12 | 1 | 0 | 0 | 0 | 0 | | 0 | | 0 | | 0 | 0 | 0 | 0 | 0 | 0 | 0 | 0 | 0 |
| ***Acidobacteria*** | 938 | 3 | 4 | 3 | 1 | 0 | | 0 | | 1 | | 0 | 0 | 0 | 0 | 0 | 0 | 1 | 1 | 2 |
| ***Actinobacteria*** |  |  |  |  |  |  | |  | |  | |  |  |  |  |  |  |  |  |  |
| *Actinobacteria* | 7894 | 170 | 112 | 98 | 51 | 42 | | 41 | | 48 | | 43 | 33 | 45 | 48 | 35 | 74 | 112 | 82 | 83 |
| ***Armatimonadetes*** | 54 | 0 | 1 | 0 | 0 | 0 | | 0 | | 0 | | 0 | 0 | 0 | 0 | 0 | 0 | 0 | 2 | 1 |
| ***Bacteroidetes*** |  |  |  |  |  |  | |  | |  | |  |  |  |  |  |  |  |  |  |
| *Bacteroidia* | 2678 | 20 | 24 | 12 | 3 | 4 | | 1 | | 1 | | 0 | 1 | 1 | 2 | 7 | 0 | 7 | 2 | 4 |
| *Flavobacteria* | 1434 | 227 | 213 | 268 | 191 | 179 | | 27 | | 22 | | 10 | 123 | 131 | 59 | 207 | 11 | 280 | 265 | 301 |
| *Sphingobacteria* | 902 | 60 | 44 | 48 | 10 | 18 | | 5 | | 3 | | 0 | 4 | 3 | 1 | 7 | 2 | 39 | 19 | 22 |
| ***BRC1*** | 12 | 1 | 0 | 0 | 0 | 0 | | 0 | | 0 | | 0 | 0 | 0 | 0 | 0 | 0 | 0 | 0 | 0 |
| ***Chlorobi*** | 113 | 1 | 0 | 0 | 0 | 0 | | 0 | | 0 | | 0 | 0 | 0 | 0 | 0 | 0 | 0 | 0 | 0 |
| ***Chloroflexi*** | 588 | 8 | 3 | 3 | 1 | 0 | | 0 | | 0 | | 0 | 0 | 0 | 0 | 0 | 0 | 0 | 0 | 0 |
| ***Cyanobacteria*** | 820 | 1 | 0 | 1 | 0 | 1 | | 0 | | 0 | | 0 | 0 | 0 | 0 | 1 | 0 | 0 | 0 | 0 |
| ***Fibrobacteres*** | 26 | 0 | 0 | 0 | 0 | 0 | | 0 | | 0 | | 0 | 0 | 0 | 0 | 0 | 0 | 1 | 0 | 0 |
| ***Firmicutes*** |  |  |  |  |  |  | |  | |  | |  |  |  |  |  |  |  |  |  |
| *Bacilli* | 5946 | 136 | 52 | 58 | 1 | 1 | | 2 | | 4 | | 1 | 1 | 2 | 1 | 1 | 12 | 8 | 3 | 17 |
| *Clostridia* | 13427 | 168 | 97 | 117 | 1 | 0 | | 0 | | 1 | | 0 | 0 | 0 | 0 | 0 | 3 | 57 | 3 | 13 |
| ***Gemmatimonadetes*** | 171 | 11 | 6 | 7 | 1 | 1 | | 0 | | 1 | | 1 | 1 | 1 | 1 | 0 | 2 | 1 | 1 | 1 |
| ***GN04*** | 14 | 2 | 1 | 2 | 1 | 0 | | 0 | | 1 | | 0 | 0 | 0 | 0 | 0 | 0 | 2 | 0 | 1 |
| ***HDBW-WB69*** | 3 | 0 | 0 | 0 | 0 | 0 | | 0 | | 0 | | 0 | 0 | 0 | 0 | 0 | 0 | 1 | 0 | 0 |
| ***Lentisphaerae*** | 36 | 0 | 0 | 0 | 0 | 0 | | 0 | | 0 | | 0 | 0 | 0 | 0 | 0 | 0 | 0 | 1 | 1 |
| ***Nitrospirae*** | 121 | 0 | 1 | 0 | 0 | 0 | | 0 | | 0 | | 0 | 0 | 0 | 0 | 0 | 0 | 0 | 0 | 0 |
| ***OP11*** | 20 | 1 | 1 | 1 | 0 | 0 | | 0 | | 0 | | 0 | 0 | 0 | 0 | 0 | 0 | 0 | 0 | 0 |
| ***OP9*** | 30 | 2 | 0 | 0 | 0 | 0 | | 0 | | 0 | | 0 | 0 | 0 | 0 | 0 | 0 | 0 | 0 | 0 |
| ***Planctomycetes*** | 500 | 15 | 7 | 6 | 1 | 0 | | 0 | | 1 | | 0 | 0 | 1 | 1 | 1 | 0 | 9 | 5 | 5 |
| ***Proteobacteria*** |  |  |  |  |  |  | |  | |  | |  |  |  |  |  |  |  |  |  |
| *Alphaproteobacteria* | 4730 | 250 | 107 | 138 | 28 | 9 | | 6 | | 11 | | 15 | 14 | 13 | 29 | 17 | 86 | 83 | 56 | 70 |
| *Betaproteobacteria* | 4661 | 726 | 626 | 818 | 327 | 409 | | 216 | | 137 | | 117 | 149 | 193 | 164 | 167 | 201 | 908 | 514 | 501 |
| *Deltaproteobacteria* | 1616 | 11 | 8 | 9 | 3 | 3 | | 2 | | 2 | | 2 | 1 | 1 | 2 | 2 | 1 | 9 | 18 | 12 |
| *Epsilonproteobacteria* | 598 | 0 | 8 | 7 | 0 | 0 | | 0 | | 0 | | 0 | 0 | 0 | 0 | 0 | 0 | 0 | 0 | 0 |
| *Gammaproteobacteria* | 7875 | 345 | 352 | 279 | 507 | 229 | | 333 | | 343 | | 269 | 472 | 426 | 259 | 277 | 436 | 581 | 137 | 393 |
| ***SAR406*** | 68 | 0 | 0 | 0 | 0 | 0 | | 0 | | 2 | | 0 | 0 | 0 | 0 | 0 | 0 | 1 | 1 | 1 |
| ***SPAM*** | 17 | 1 | 0 | 0 | 0 | 0 | | 0 | | 0 | | 0 | 0 | 0 | 0 | 0 | 0 | 0 | 0 | 0 |
| ***Spirochaetes*** | 487 | 1 | 0 | 0 | 0 | 0 | | 0 | | 0 | | 0 | 0 | 0 | 0 | 0 | 0 | 0 | 2 | 0 |
| ***SR1*** | 6 | 0 | 0 | 0 | 0 | 0 | | 0 | | 0 | | 0 | 0 | 0 | 0 | 0 | 0 | 1 | 0 | 0 |
| ***Synergistetes*** | 75 | 7 | 2 | 2 | 0 | 0 | | 0 | | 0 | | 0 | 0 | 0 | 0 | 0 | 0 | 0 | 0 | 0 |
| ***Tenericutes*** | 880 | 3 | 2 | 2 | 0 | 0 | | 0 | | 0 | | 0 | 0 | 0 | 0 | 0 | 0 | 0 | 0 | 1 |
| ***TG3*** | 12 | 0 | 2 | 0 | 0 | 0 | | 0 | | 0 | | 0 | 0 | 0 | 0 | 0 | 0 | 0 | 0 | 0 |
| ***Thermi*** | 110 | 2 | 0 | 1 | 0 | 0 | | 0 | | 0 | | 0 | 0 | 0 | 1 | 1 | 0 | 1 | 0 | 0 |
| ***Thermodesulfobacteria*** | 12 | 2 | 0 | 0 | 0 | 0 | | 0 | | 0 | | 0 | 0 | 0 | 0 | 0 | 0 | 0 | 0 | 0 |
| ***TM7*** | 67 | 5 | 6 | 1 | 1 | 0 | | 0 | | 0 | | 0 | 0 | 0 | 0 | 1 | 1 | 2 | 2 | 2 |
| ***Verrucomicrobia*** | 625 | 19 | 25 | 5 | 1 | 0 | | 0 | | 0 | | 0 | 0 | 0 | 0 | 0 | 1 | 3 | 4 | 4 |
| ***WPS-2*** | 20 | 1 | 0 | 0 | 0 | 0 | | 0 | | 0 | | 0 | 0 | 0 | 0 | 0 | 0 | 0 | 0 | 0 |
| Total |  | 2200 | 1704 | 1886 | 1129 | 896 | | 633 | | 578 | | 458 | 799 | 817 | 568 | 724 | 830 | 2107 | 1118 | 1435 |

^*^The major phyla, *Actinobacteria*, *Bacteroidetes*, *Firmicutes*, and *Proteobacteria* were further refined into the class level. All laboratory cultures were inoculated with April 2012 sludge sample. An OTU was counted if it was called present in at least 2 of 3 replicates in all samples except [C2mim]Cl, No-Carbon_F, No-Carbon_R1, and No-carbon_R2 samples, which show data from a single PhyloChip (AB: aeration basin sample, _F: flask cultures, _R: reactor cultures).
